# CD45RO^+^记忆T细胞作为非小细胞肺癌患者预后标志物的研究

**DOI:** 10.3779/j.issn.1009-3419.2021.103.05

**Published:** 2021-04-20

**Authors:** 琪凡 谭, 浩洋 李, 孟军 俞, 晓楠 唐, 金晶 谭, 树才 张, 敬慧 王

**Affiliations:** 1 101149 北京，首都医科大学附属北京胸科医院肿瘤内科 Department of Medical Oncology, Beijing Chest Hospital, Capital Medical University, Beijing Tuberculosis and Thoracic Tumor Research Institute, Beijing 101149, China; 2 101149 北京，首都医科大学附属北京胸科医院北京市结核病胸部肿瘤研究所肿瘤研究中心 Cancer Research Center, Beijing Chest Hospital, Capital Medical University, Beijing Tuberculosis and Thoracic Tumor Research Institute, Beijing 101149, China

**Keywords:** 肺肿瘤, CD45RO, 程序性死亡受体-配体1, 预后, Lung neoplasms, CD45RO, Programmed cell death ligand 1, Prognosis

## Abstract

**背景与目的:**

肺癌是世界范围内最常见的恶性肿瘤之一。肿瘤微环境中多种多样的免疫浸润细胞，是肿瘤免疫的重要组成，对患者预后具有临床意义。CD45RO^+^肿瘤浸润淋巴细胞（tumor infiltrating lymphocytes, TILs），即记忆T细胞，其表达与多种肿瘤预后相关。本研究旨在探讨非小细胞肺癌（non-small cell lung cancer, NSCLC）中评估肿瘤和基质区CD45RO^+^ TILs密度与患者临床特征和预后的关系，及其联合程序性死亡受体配体1（programmed cell death-ligand 1, PD-L1）作为预后标志物的临床价值。

**方法:**

对167例NSCLC患者的组织微阵列进行多重荧光免疫组织化学染色，标记CD45RO、细胞角蛋白（cytokeratin, CK）和PD-L1。利用人工智能图像识别技术和肿瘤细胞特异性CK染色，划分组织中的肿瘤区和基质区，评估肿瘤区和基质区CD45RO^+^ TILs的密度以及肿瘤细胞的PD-L1表达水平。采用非参检验分析CD45RO^+^ TILs与患者临床特征的关系，使用*Kaplan-Meier*方法和*Cox*风险比例模型分析CD45RO^+^ TILs独立或与PD-L1联合与肿瘤预后的关系。

**结果:**

CD45RO^+^ TILs的密度与患者年龄、吸烟、肿瘤分期和病理类型显著相关。在NSCLC和肺腺癌（lung adenocarcinoma, LUAD）患者中，基质区高密度CD45RO^+^ TILs具有更长的总生存期（overall survival, OS）（NSCLC: *P*=0.007; LUAD: *P* < 0.001），并且是OS的独立预后因素（NSCLC: HR=0.559, 95%CI: 0.377-0.829, *P*=0.004; LUAD: HR=0.352, 95%CI: 0.193-0.641, *P*=0.001）。联合肿瘤细胞的PD-L1评分以及所有区域CD45RO^+^ TILs的浸润评分将患者分为四组：其中PD-L1^+^/CD45RO^+^患者无病生存期（disease-free survival, DFS）最长，PD-L1^+^/CD45RO^-^的患者DFS时间最短，并可作为DFS预后的独立因素（HR=2.221, 95%CI: 1.258-3.919, *P*=0.006）。

**结论:**

肿瘤组织中CD45RO^+^ TILs密度以及CD45RO^+^ TILs联合肿瘤区PD-L1，与NSCLC的临床病理特征及预后显著相关，可作为新的生存预后标志物。

## Abstract (en)

**Background and objective:**

Lung cancer is the most common malignancy world-wide. There are a variety of immune infiltrating cells in tumor microenvironment, which is an important component of tumor immunity and has clinical significance for the prognosis of patients. CD45RO is a surface marker of memory T cells. The expression of CD45RO^+^ tumor infiltrating lymphocytes (TILs) is associated with the prognosis of many tumors. The purpose of this study was to evaluate the relationship between the density of CD45RO^+^ TILs in tumor and stromal area and the clinical characteristics of patients with non-small cell lung cancer (NSCLC) and its impact on the prognosis of patients. We aimed to explore the clinical value of CD45RO^+^ TILs and programmed cell death ligand 1 (PD-L1) as prognostic markers.

**Methods:**

Multiple fluorescent immunohistochemical staining was used to stain the tissue microarray chips of 167 patients with NSCLC, marking CD45RO, cytokeratin (CK) and PD-L1. Using artificial intelligence image recognition technology and tumor cell-specific CK staining, divide the tumor and stromal area in the tissue, evaluate the density of CD45RO^+^ TILs in the tumor and stromal area, and the expression level of PD-L1 in tumor cells. The non-parametric test was used to analyze the relationship between CD45RO^+^ TILs and the clinical characteristics of patients, and the *Kaplan-Meier* method and *Cox* risk ratio model were used to analyze the relationship between CD45RO^+^ TILs independently or in combination with PD-L1 and tumor prognosis.

**Results:**

The density of CD45RO^+^ TILs was significantly associated with patient age, smoking, tumor stage, and pathological type. Single-factor survival analysis showed that NSCLC (*P*=0.007) stromal region and lung adenocarcinoma (LUAD) (*P* < 0.001) with CD45RO^+^ TILs high density had better OS. Multivariate survival analysis showed that the high density of CD45RO^+^ TILs in the stromal region of NSCLC (HR=0.559, 95%CI: 0.377-0.829, *P*=0.004) and lung adenocarcinoma (HR=0.352, 95%CI: 0.193-0.641, *P*=0.001) were independent prognostic factors for overall survival time (OS). Combined with PD-L1 score of tumor cells in tumor tissues and infiltration score of CD45RO^+^ TILs in all tumor tissues, the patients were divided into 4 groups: patients with PD-L1^+^/CD45RO^+^ had the longest disease-free survival (DFS) time, and patients with PD-L1^+^/CD45RO^-^ had the shortest DFS time. Multivariate *Cox* regression analysis showed that PD-L1^+^/CD45RO^-^ was an independent prognostic factor for DFS and had a higher risk of poor prognosis compared to the other three groups (HR=2.221, 95%CI: 1.258-3.919, *P*=0.006).

**Conclusion:**

In tumor tissues, the density of CD45RO^+^ TILs, as well as the combination of CD45RO^+^ TILs and PD-L1 in tumor areas, significantly correlated with clinicopathological features and prognosis of NSCLC, which can be used as a new prognosis marker.

在世界范围内，肺癌是最常见的恶性肿瘤之一，也是导致恶性肿瘤死亡的主要原因。非小细胞肺癌（non-small cell lung cancer, NSCLC）约占原发性肺癌的85%^[1]^，70%的患者在确诊时已处于晚期，5年生存率低于15%^[2]^。传统的化疗、放疗和靶向治疗等综合治疗可显著延长患者生存期。但化疗的中位生存期仅8个月-10个月，靶向治疗不可避免的会出现耐药^[3]^。随着肿瘤学、免疫学、分子生物学等相关学科的迅速发展，免疫治疗给肿瘤治疗带来了革命性的变化，被认为是最有希望治愈恶性肿瘤的治疗手段。免疫检查点抑制剂（immune checkpoint inhibitors, ICIs），包括Pembrolizumab、Nivolumab、Atezolizumab和Durvolumab已被美国食品药品管理局（Food and Drug Administration, FDA）批准用于治疗肺癌^[4-7]^。然而，只有约20%的NSCLC患者适合ICIs，临床实践中程序性死亡受体-配体1（programmed cell death ligand 1, PD-L1）表达和肿瘤突变负担的预测作用不足^[8]^。为了改善NSCLC患者的预后，迫切需要发现新的免疫生物标志物。

通过揭示肿瘤微环境（tumor microenvironment, TME）在控制肿瘤行为中的关键作用^[9, 10]^，肿瘤浸润淋巴细胞（tumor infiltrating lymphocytes, TILs）作为TME的重要组成部分的预后意义及其可能的预测价值已成为癌症研究的热点。研究表明，TILs在头颈部^[11]^、肺部^[12]^、肾脏^[13]^等多种癌症的预后中具有重要意义。一些研究报道了TILs的存在与预后之间的正相关关系^[14]^，而其他研究报道了没有关系^[15]^或负相关关系^[16]^。

人类CD45是一种跨膜酪氨酸磷酸酶，存在于除红细胞外的所有造血细胞上。它们以不同的亚型存在，这些亚型在指定为CD45R的一组限制性细胞类型上表达^[17]^。大多数初始T细胞表达一种称为CD45RA的CD45R，而记忆T细胞表达另一种亚型，称为CD45RO^[18]^。CD45RO^+^ T淋巴细胞与初始T细胞（CD45RA^+^）相比，遇到抗原反应更快，抗原刺激强度更大^[19]^。现已有多项研究报道了，CD45RO^+^ TILs的高表达与癌症较好的预后显著相关，包括食管癌^[20, 21]^、结直肠癌^[22, 23]^、乳腺癌^[24]^、子宫内膜癌^[25]^、胃癌^[26]^和肝细胞癌^[27]^等。在一项结直肠癌的研究中发现，CD45RO^+^ TILs也可被用作潜在的NSCLC的候选免疫标志物^[28]^。因此，我们拟研究CD45RO^+^ TILs细胞对NSCLC患者预后的影响。本研究评估了CD45RO^+^ TILs密度对Ⅰ期-Ⅲ期NSCLC患者生存预后的影响及其与患者临床特征的关系。PD-L1与患者的预后^[29]^息息相关，PD-L1过表达与亚洲NSCLC患者总生存期（overall survival, OS）时间更短有关。本研究还联合CD45RO^+^ TILs密度和患者肿瘤细胞PD-L1表达水平，探索双标志物联用对NSCLC患者的预后价值。

## 1 材料与方法

### 1.1 一般资料

回顾性地纳入2013年1月-2016年12月在首都医科大学附属北京胸科医院接受治疗的患者，纳入标准：①新诊断的原发性NSCLC患者；②术前未接受治疗；③充分的随访信息。排除标准：①我们没有足够组织进行检测的患者；②有其他恶性肿瘤的患者。肿瘤根据美国癌症联合会（American Joint Committee on Cancer, AJCC）指南（第8版）进行分期。根据世界卫生组织指南进行亚型分类^[30]^。患者手术切除的石蜡包埋组织制备成组织微阵列（tissue microarray, TMA）。本研究经首都医科大学附属北京胸科医院伦理委员会批准。

167例NSCLC患者中位年龄61岁（35岁-84岁），其中96例患者（57.5%） > 60岁，男性119例（71.3%），女性48例（28.7%）。167例患者中，吸烟患者103例（61.7%）。其分期为Ⅰ期48例（28.8%），Ⅱ期42例（25.1%），Ⅲ期77例（46.1%）。其他肿瘤特征为淋巴结转移阳性81例（48.5%）、淋巴结转移阴性86例（51.5%）；肺鳞状细胞癌（lung squamous cell carcinoma, LUSC）77例（46.1%）和肺腺癌（lung adenocarcinoma, LUAD）90例（53.9%）。

### 1.2 多重荧光免疫组化染色

使用Opal 7色多重荧光染色试剂（PerkinElmer, USA）在4 μm厚的TMA切片上进行染色。免疫组化（immunohistochemistry, IHC）法对TMA切片进行脱蜡和水化后，用柠檬酸盐进行组织抗原修复。此后每一步标记染色步骤根据试剂盒说明书进行，简述为：用阻断缓冲液阻断25 min后，首先在室温下与一抗孵育2 h，然后在室温下与二抗（Opal Polymer HRP Ms+Rb, USA）孵育30 min，然后洗涤；为了产生荧光信号，TMA切片在室温下与Opal工作液孵育10 min。在下一个标记染色之前，切片用微波炉加热，去除非共价结合的抗体，之后重复上述步骤。最后用DAPI染色细胞核，封片^[31]^。使用的一抗为：细胞角蛋白CK单克隆抗体（pan-CK cocktail, Abcam）、CD45RO蛋白单克隆抗体（UCHL1克隆，ThermoFisher）、PD-L1单克隆抗体（E1L3N克隆，CST）。

### 1.3 多重荧光染色结果

使用Vectra2.1多光谱显微镜成像系统（PerkinElmer，美国）采集多重荧光染色组织切片图像。使用Inform 2.2软件（PerkinElmer，美国）对组织切片图像进行光谱拆分；组织类型拆分；细胞识别；细胞分型；计数评分，从而对图像进行分析。使用DAPI细胞核染色进行细胞识别。样本中每个标记物的表达都是通过密度来分析的，密度根据阳性细胞的百分比来计算。

### 1.4 统计分析

采用非参检验分析CD45RO^+^ TILs与患者临床特征的关系，使用*Kaplan-Meier*方法和*Cox*风险比例模型分析其与预后和临床病理特征的关系。统计分析和作图使用SPSS（IBM，美国）和R（3.6.5）进行。

## 2 结果

### 2.1 NSCLC组织芯片多重荧光免疫组化染色

我们在167例NSCLC肿瘤组织中检测了CK、CD45RO以及PD-L1蛋白的表达情况（图 1）。患者人口学特征见表 1。CK标记为绿色，特异表达在肿瘤细胞质。CD45RO标记为红色，表达在T淋巴细胞膜表面。PD-L1标记为黄色，表达在细胞膜表面。利用人工智能图像识别技术，结合细胞形态和CK蛋白的染色情况，我们将组织划分为肿瘤区和基质区。肿瘤区主要由癌巢的肿瘤细胞以及浸润的淋巴细胞构成。基质区内的主要组成为成纤维细胞、内皮细胞、淋巴细胞等。

**图 1:**
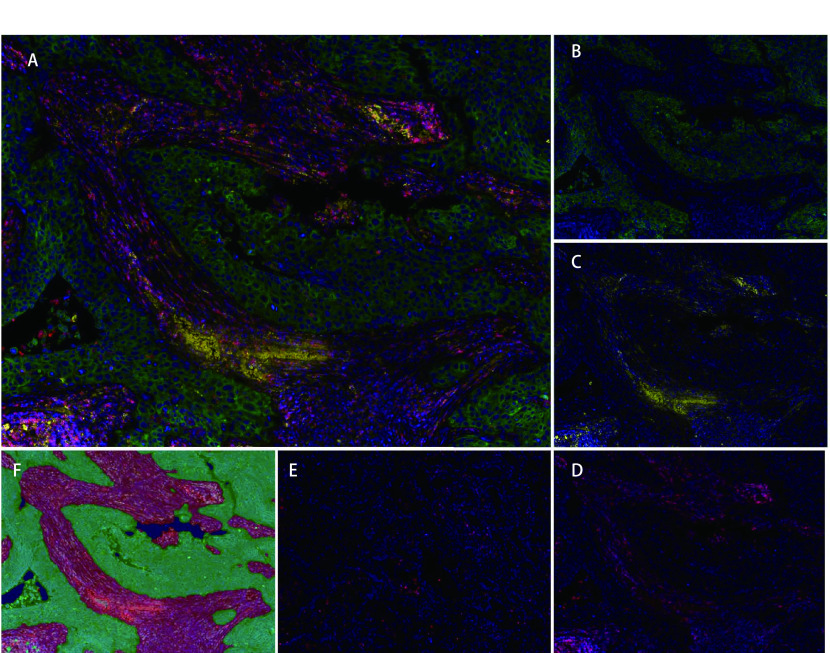
多标记免疫荧光染色后CD45RO和PD-L1在非小细胞肺癌中的表达。A：CD45RO、PD-L1、CK表达及DAPI染色的共定位图片（×200）；B：CK表达（细胞质，绿色）（×200）；C：PD-L1表达（细胞膜，黄色）（×200）；D：CD45RO^+^表达（细胞膜，红色）（×200）；E：CD45RO^-^表达（T淋巴细胞膜，红色）（×200）；F：肿瘤区和基质区依照组织细胞形态和CK表达情况进行划分：肿瘤区（绿色），基质区（红色）（×200） CD45RO and PD-L1 expression in non-small cell lung cancer by multiplex immunofluorescence staining. A: a merged picture of expression of CD45RO, PD-L1, CK and DAPI (×200); B: CK expression (cytoplasmic, Green) (×200); C: PD-L1 expression (Cell membrane, Yellow) (×200); D: CD45RO^+^ expression (Cell membrane, Red) (×200); E: CD45RO^-^ expression (Cell membrane, Red) (×200); F: tumor compartments and stromal compartments divided by CK, Tumor (Green), stromal (Red) (×200)

**表 1:** 患者的临床特征 Patients' characteristics

Clinical features	*n*	Percentage
Gender		
Male	119	71.3%
Female	48	28.7%
Smoking history		
Yes	103	61.7%
No	64	38.3%
Age (yr)		
Median	61	
≤60	71	42.5%
> 60	96	57.5%
TNM stage		
Ⅰ	48	28.8%
Ⅱ	42	25.1%
Ⅲ	77	46.1%
Lymphatic metastasis		
Positive	81	48.5%
Negative	86	51.5%
Histology		
Adenocarcinoma	90	53.9%
Squamous	77	46.1%
TNM: tumor-node-metastasis		

### 2.2 CD45RO^+^ TILs的密度与临床病理特征的关系

CD45RO^+^ TILs在肿瘤区和基质区均有富集，基质区的密度较肿瘤区更高。我们分别针对肿瘤区和基质区的CD45RO^+^ TILs，分析其密度与患者性别、年龄、吸烟情况、临床分期等临床特征之间的关系。在NSCLC肿瘤区， > 60岁患者CD45RO^+^ TILs密度高于≤60岁患者（*P*=0.027），吸烟患者CD45RO^+^ TIL密度高于非吸烟患者（*P*=0.006），Ⅱ期患者CD45RO^+^ TIL密度高于Ⅰ期和Ⅲ期患者（*P*=0.014）。基质区CD45RO^+^ TILs的密度在各临床特征分层之间的差异无统计学意义。进一步对肺癌病理亚型分析发现，肺鳞癌患者CD45RO^+^ TILs密度高于肺腺癌患者（图 2），无论是在肿瘤区（*P*=0.039）还是在基质区（*P*=0.002）。吸烟与非吸烟患者肿瘤区CD45RO^+^ TILs密度的差异主要体现在肺腺癌患者中（*P*=0.005）。而Ⅱ期患者肿瘤区CD45RO^+^ TILs相较于Ⅰ期和Ⅲ期的高度富集则体现在肺鳞癌患者中（*P*=0.027）（图 3）。

**图 2:**
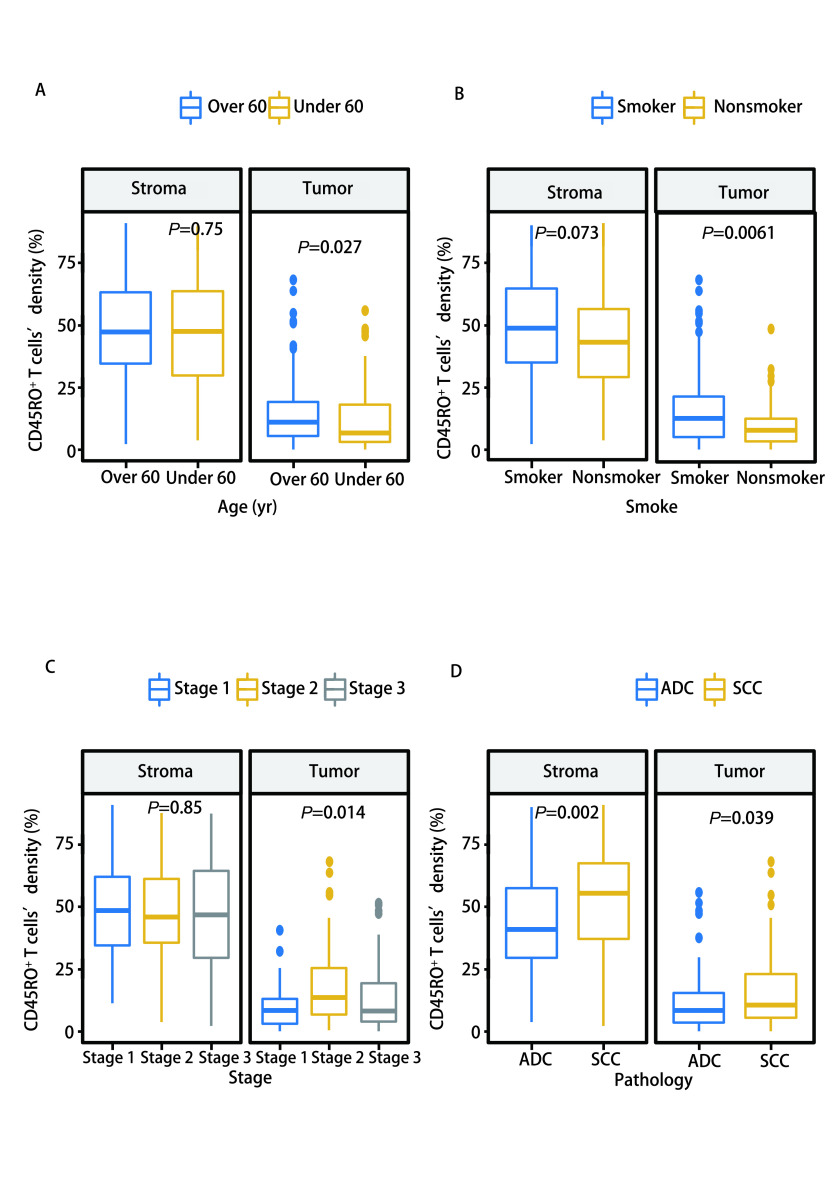
非小细胞肺癌中CD45RO^+^ TILs与临床病理特征的关系。A：根据年龄；B：根据吸烟；C：根据临床分期；D：根据病理类型。 Significance of CD45RO^+^ TILs with clinicopathologic characteristics in non-small cell lung cancer. A: according to age; B: according to smoking; C: according to clinical stage; D: according to pathological type. TILs: tumor infiltrating lymphocytes; ADC: lung adenocarcinoma; SCC: lung squamous cell carcinoma

**图 3:**
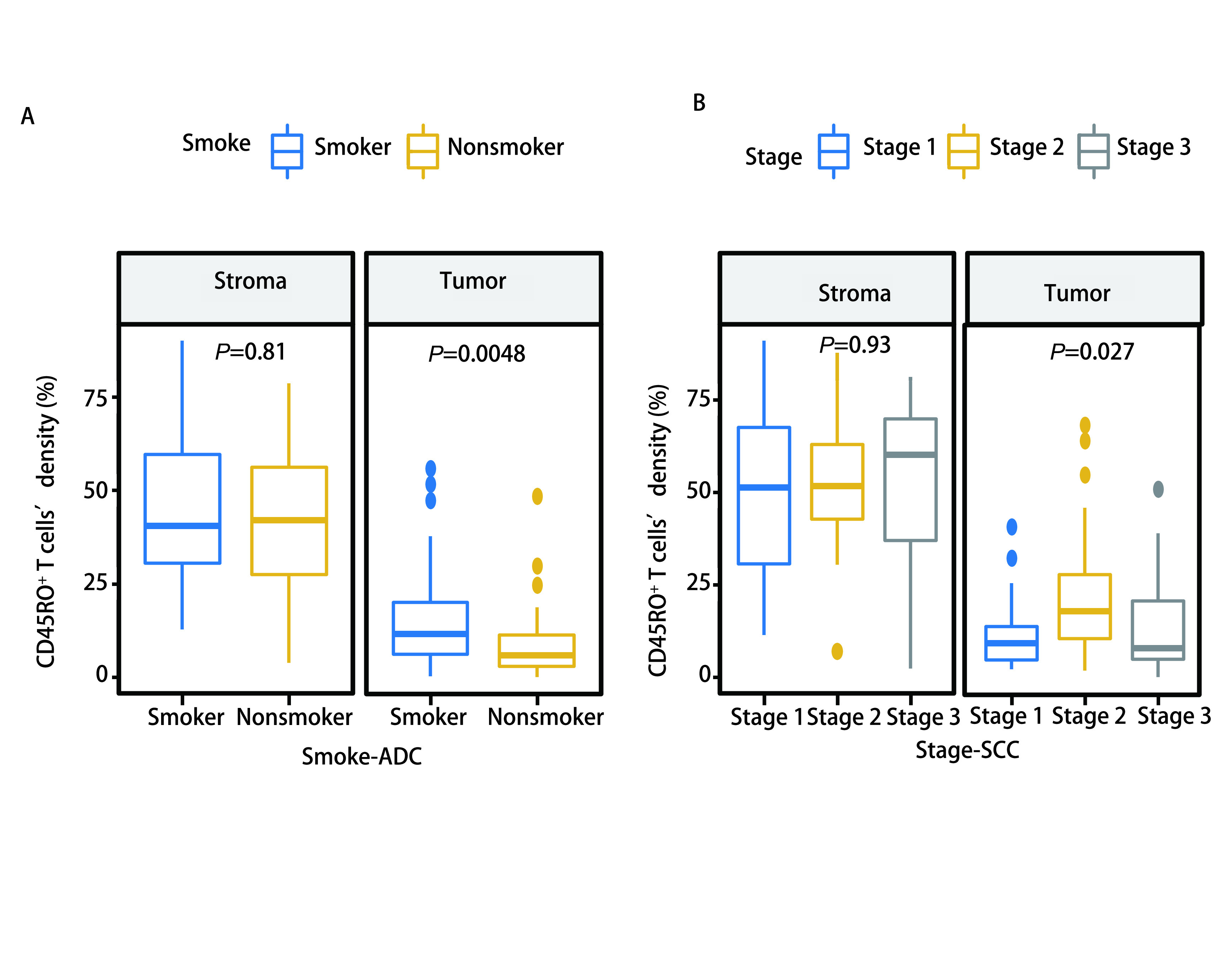
肺腺癌及肺鳞癌中CD45RO^+^ TILs与临床病理特征的关系。A：肺腺癌中CD45RO^+^ TILs密度与吸烟的关系；B：肺鳞癌CD45RO^+^ TILs密度与临床分期的关系 Relationship between CD45RO^+^ TILs and clinicopathological features in lung adenocarcinoma and lung squamous cell carcinoma. A: relationship between CD45RO^+^ TILs density and smoking in lung adenocarcinoma; B: relationship between CD45RO^+^ TILs density and clinical stage in lung squamous cell carcinoma.

### 2.3 CD45RO^+^ TILs的密度与患者的生存关系

所有患者的最后一次随访日期为2019年6月30日。当时，已有105例患者死亡，62例患者存活。随访记录了患者疾病复发和死亡的日期，计算出患者无病生存期（disease-free survival, DFS）和OS。生存分析发现在NSCLC肿瘤组织基质区CD45RO^+^ TILs的密度与患者OS显著相关（*P*=0.007），浸润程度高的患者预后生存时间较长（图 4）。多因素*Cox*回归分析显示，基质区CD45RO^+^ TILs的密度是NSCLC的独立预后因素（HR=0.559, 95%CI: 0.377-0.829, *P*=0.004）（表 2）。由于CD45RO^+^ TILs在肺鳞癌和肺腺癌之间表达差异较大，我们对肺鳞癌和肺腺癌分别进行了生存分析。在肺腺癌患者中，基质区CD45RO^+^ TILs密度与患者的DFS和OS都密切相关，CD45RO^+^ TILs密度高的患者都有更长的生存时间（DFS: *P*=0.034; OS: *P* < 0.001）。多因素*Cox*回归分析显示，基质区CD45RO^+^ TILs的密度是肺腺癌OS的独立预后因素（HR=0.352, 95%CI: 0.193-0.641, *P*=0.001）（表 3）。在肺鳞癌患者中，尽管有相同的趋势，但结果没有获得统计学意义。而肿瘤区的CD45RO^+^ TILs的密度无论是在鳞癌还是腺癌中，都与患者的DFS和OS均没有显著相关性。

**图 4:**
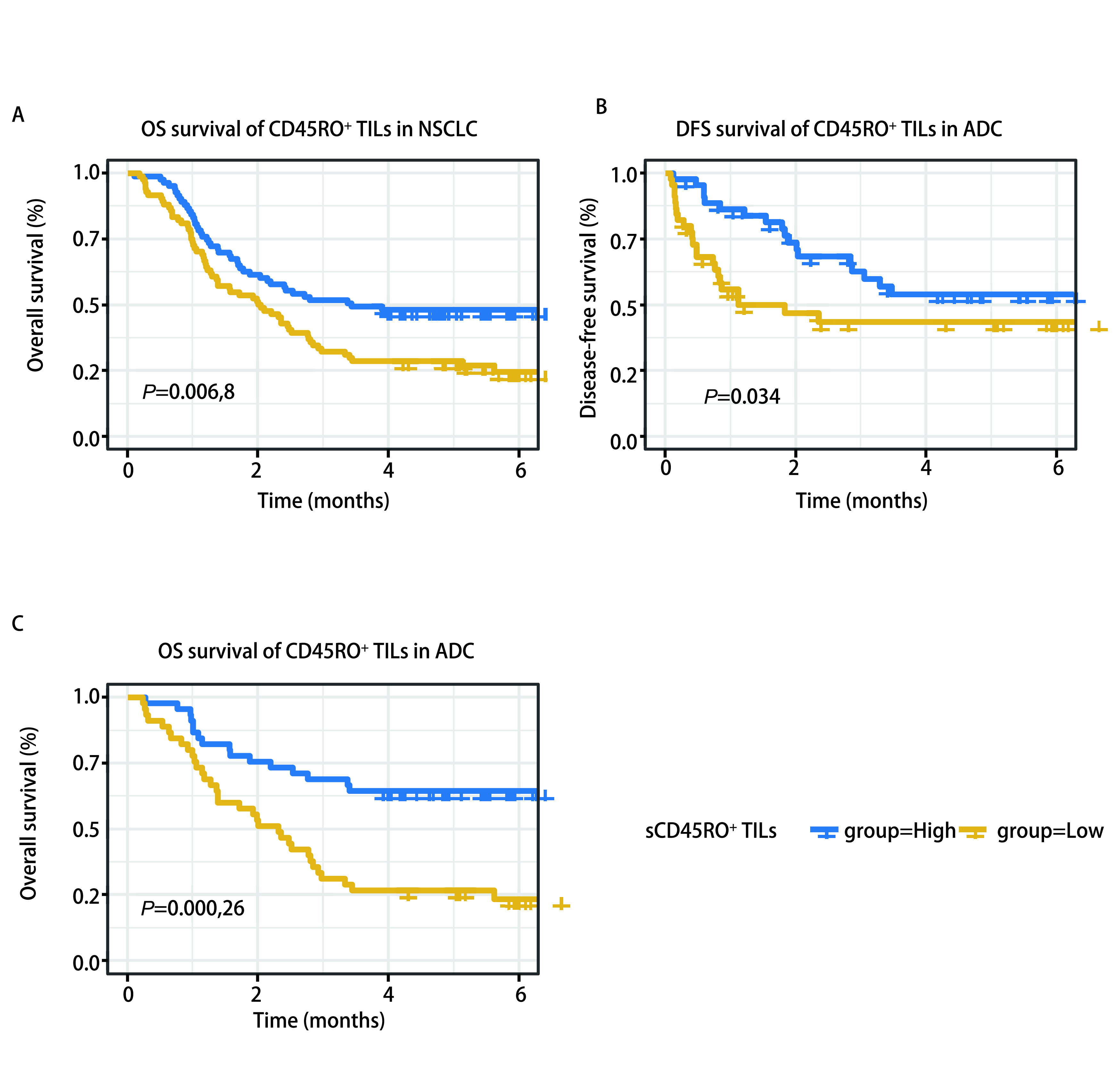
CD45RO^+^ TILs与无病生存期以及总生存期的*Kaplan-Meier*曲线分析。A：非小细胞肺癌基质区CD45RO^+^ TILs密度与患者总生存期显著相关；B：肺腺癌基质区CD45RO^+^ TILs密度与患者无病生存期密切相关；C：肺腺癌基质区CD45RO^+^ TILs密度与患者总生存期密切相关。 *Kaplan-Meier* curves for Correlations of CD45RO^+^ TILs with disease-free survival and overall survival. A: the density of CD45RO^+^ TILs in the stromal compartment of non-small cell lung cancer was significantly associated with patient overall survival; B: the density of CD45RO^+^ TILs in the stromal compartment of lung adenocarcinoma was strongly associated with patient disease-free survival; C: the density of CD45RO^+^ TILs in the stromal region of lung adenocarcinoma was strongly associated with patients' OS; OS: overall survival; DFS: disease-free survival.

**表 2:** 多因素分析CD45RO^+^ TILs在非小细胞肺癌中的总生存期 Multivariate analysis of CD45RO^+^ TILs in non-small cell lung cancer overall survival

	HR	95%CI	*P*
Smoke (Yes *vs* No)	1.215	0.809-1.826	0.348
Stage (Ⅰ+Ⅱ *vs* Ⅲ)	0.443	0.246-0.797	0.007
Stroma CD45RO (High *vs* Low)	0.559	0.377-0.829	0.004
Node metastasis (1=Positive; 0=Negative)	1.023	0.571-1.833	0.940

**表 3:** 多因素分析CD45RO^+^ TILs在肺腺癌中的总生存期 Multivariate analysis of overall survival of CD45RO^+^ TILs in lung adenocarcinoma

	HR	95%CI	*P*
Smoke (Yes *vs* No)	1.125	0.643-1.967	0.68
Stage (Ⅰ+Ⅱ *vs* Ⅲ)	0.397	0.160-0.984	0.046
Stroma CD45RO (High *vs* Low)	0.352	0.193-0.641	0.001
Node metastasis (1=Positive; 0=Negative)	0.911	0.371-2.236	0.838

### 2.4 CD45RO^+^ TILs联合PD-L1表达与患者的生存关系

目前的研究报道显示，肿瘤细胞高表达PD-L1能抑制效应T细胞对肿瘤细胞的杀伤作用，是影响患者预后的一个重要因素。因此我们将肿瘤组织中所有区域CD45RO^+^ TILs的密度联合肿瘤细胞表达PD-L1的评分将患者分为四组：PD-L1^+^/CD45RO^+^，标志物双阳性组；PD-L1^-^/CD45RO^-^，标志物双阴性组；PD-L1^+^/CD45RO^-^和PD-L1^-^/CD45RO^+^，标志物单阳性组。

这四组NSCLC患者的DFS（*P*=0.021）和OS（*P*=0.031）生存曲线呈现明显阶梯差异，其中标志物双阳性患者DFS及OS时间最长，PD-L1^+^/CD45RO^-^的患者最短（图 5A、图 5B）。多因素*Cox*回归分析结果显示，PD-L1^+^/CD45RO^-^是DFS（HR=2.221, 95%CI: 1.258-3.919, *P*=0.006）（表 4）及OS（HR=1.781, 95%CI: 1.114-2.845, *P*=0.016）（表 5）预后的独立因素，相较于其他三组患者预后差的风险更高。在肺腺癌中，PD-L1^-^/CD45RO^+^组较其他三组有更好的OS（*P*=0.004，图 5C），多因素*Cox*回归分析结果显示，PD-L1^-^/CD45RO^+^是OS（HR=0.446, 95%CI: 0.225-0.885, *P*=0.021）预后的独立因素（表 6）。

**图 5:**
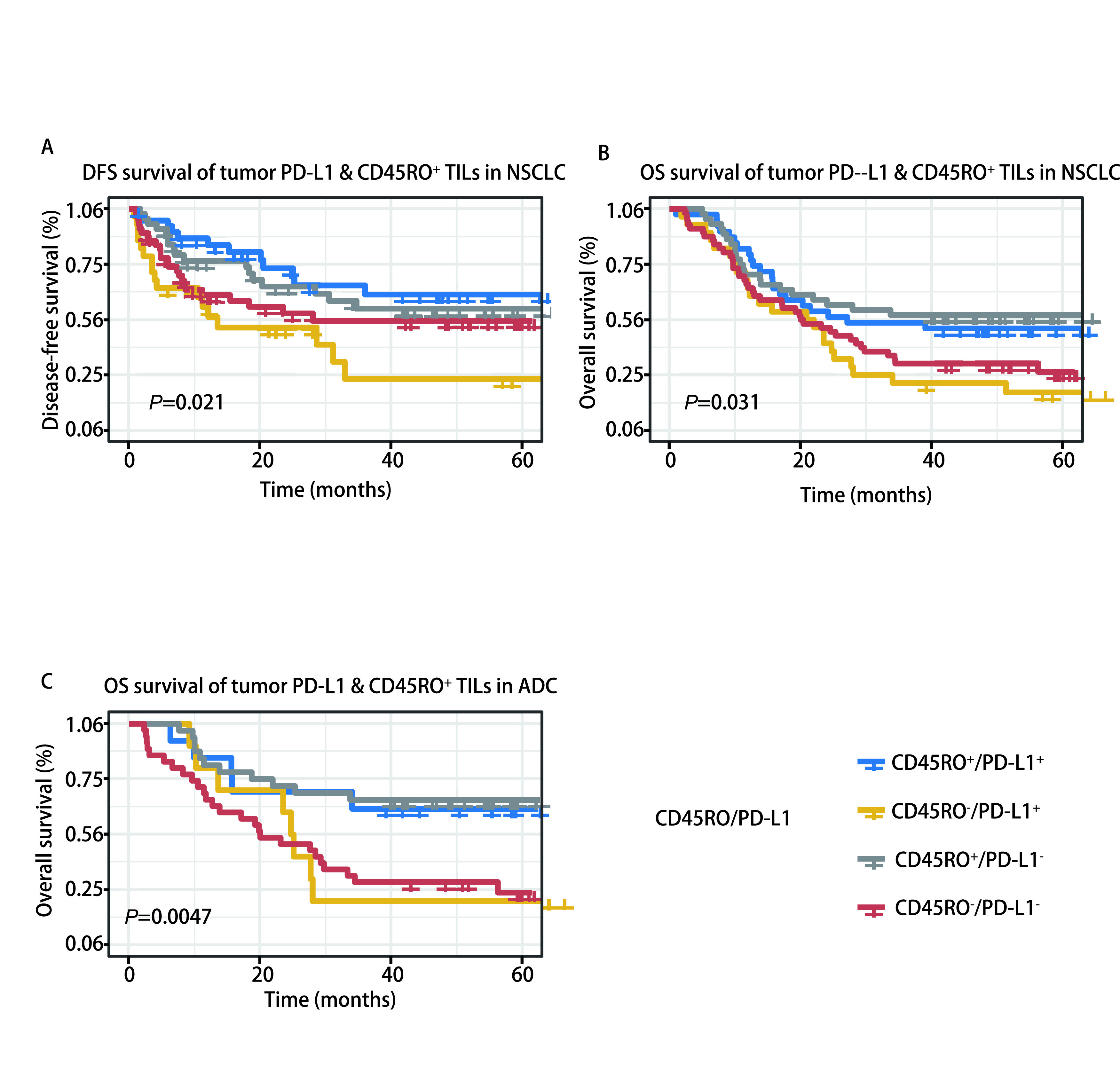
CD45RO^+^ TILs联合PD-L1的表达与无病生存期以及总生存期的*Kaplan-Meier*分析。A：PD-L1^+^/CD45RO^+^组非小细胞肺癌患者无病生存期最长，PD-L1^+^/CD45RO^-^组最短；B：PD-L1^+^/CD45RO^+^组非小细胞肺癌患者总生存期最长，PD-L1^+^/CD45RO^-^组最短；C：在肺腺癌中，PD-L1^-^/CD45RO^+^组较其他三组有更好的总生存期 *Kaplan-Meier* curves for correlations of CD45RO^+^ TILs and PD-L1 expression with disease-free survival and overall survival. A: patients with non-small cell lung cancer in the PD-L1^+^/CD45RO^+^ group had the longest disease-free survival and those in the PD-L1^+^/CD45RO^-^ group had the shortest; B: non-small cell lung cancer patients in the PD-L1^+^/CD45RO^+^ group had the longest overall survival and those in the PD-L1^+^/CD45RO^-^ group had the shortest overall survival; C: in lung adenocarcinoma, the PD-L1^-^/CD45RO^+^ group had better overall survival than the other three groups

**表 4:** 多因素分析CD45RO^+^ TILs和PD-L1双标记在非小细胞肺癌中的无病生存期 Multivariate analysis of CD45RO^+^ TILs and PD-L1 double markers for disease-free survival in non-small cell lung cancer

	HR	95%CI	*P*
Smoke (Yes *vs* No)	0.808	0.499-1.309	0.387
Stage (Ⅰ+Ⅱ *vs* Ⅲ)	0.561	0.279-1.130	0.106
Combine CD45RO+PD-L1 (Group 2 *vs* Others) ^*^	2.221	1.258-3.919	0.006
Node metastasis (1=Positive; 0=Negative)	1.705	0.846-3.44	0.136
^*^Group 2=PD-L1^+^/CD45RO^-^; PD-L1: programmed cell death ligand 1			

**表 5:** 多因素分析CD45RO^+^ TILs和PD-L1双标记在非小细胞肺癌中的总生存期 Multivariate analysis of CD45RO^+^ TILs and PD-L1 double markers for overall survival in non-small cell lung cancer

	HR	95%CI	*P*
Smoke (Yes *vs* No)	1.118	0.744-1.679	0.592
Stage (Ⅰ+Ⅱ *vs* Ⅲ)	0.439	0.251-0.768	0.004
Combine CD45RO+PD-L1 (Group 2 *vs* Others) ^*^	1.781	1.114-2.845	0.016
Node metastasis (1=Positive; 0=Negative)	1.057	0.609-1.834	0.845
^*^Group2=PD-L1^+^/CD45RO^-^			

**表 6:** 多因素分析CD45RO^+^ TILs和PD-L1双标记在肺腺癌中的总生存期 Multivariate analysis of CD45RO^+^ TILs and PD-L1 double markers for overall survival in lung adenocarcinoma

	HR	95%CI	*P*
Smoke (Yes *vs* No)	0.97	0.552-1.704	0.916
Stage (Ⅰ+Ⅱ *vs* Ⅲ)	0.488	0.213-1.120	0.091
Combine CD45RO+PD-L1 (Group3 *vs* Others)^*^	0.446	0.225-0.885	0.021
Node metastasis (1=Positive; 0=Negative)	1.125	0.496-2.547	0.778
^*^Group3=PD-L1^-^/CD45RO^+^			

## 3 讨论

癌症免疫疗法的成功，已经证明免疫细胞，特别是T细胞，可以被用来消除肿瘤细胞。尽管有持续的临床疗效，但仍然只有一小部分癌症患者受益^[32]^。作为TME的主要组成部分，免疫浸润细胞已被证明对肿瘤进展以及免疫治疗反应至关重要^[33]^。因此，更好地了解TME中的先天性免疫细胞和适应性免疫细胞对于破译免疫治疗机制、定义预测性生物标志物和识别新的治疗靶点至关重要。

在肿瘤相关的免疫过程中，CD45RA^+^初始T细胞经过抗原呈递激活，分化成效应T细胞和记忆T细胞。效应T细胞包括CD4^+^辅助T细胞和CD8^+^杀伤T细胞，直接参与肿瘤杀伤过程。记忆T细胞在组织中起监视作用，能够长期存活，并且在二次免疫反应中起非常重要的作用。CD45RO为记忆T细胞的表面抗原，是区分初始T细胞和记忆T细胞的重要标志。

CD45RO^+^ T记忆细胞在NSCLC组织中的浸润情况，已有一些报道。早期研究中，研究者使用免疫组化的方法评估CD45RO^+^ TILs密度与患者临床特征和预后的关系。Paulsen等^[12]^的研究结果认为，无论是NSCLC还是肺腺癌及肺鳞癌亚组中，CD45RO^+^ TILs密度与临床病理特征的差异均无统计学意义。本研究中，我们划分了癌巢所在的肿瘤区和周围的基质区，分别分析不同浸润位置的CD45RO^+^ TILs密度与患者临床病理特征的关系。我们发现在NSCLC组织中，基质区CD45RO^+^ TILs密度较肿瘤区更高，肿瘤区CD45RO^+^ TILs密度与年龄、吸烟以及TNM分期方面差异均有统计学意义。而基质区CD45RO^+^ TILs密度与各临床特征之间的差异均无统计学意义。

同理，关于CD45RO^+^ TILs密度与NSCLC预后的关系，既往一些研究中存在的争议^[12, 34-36]^可能也是由于评价体系中没有明确CD45RO^+^ T细胞的浸润位置造成的。尹婕等^[34]^的研究中发现，CD45RO^+^ TILs在NSCLC肿瘤组织和正常组织中的表达无统计学意义。我们的研究发现基质区CD45RO^+^ TILs密度与NSCLC患者的生存预后显著相关，而肿瘤区未见显著差异。针对CD45RO^+^ TILs密度与肺腺癌和肺鳞癌关系现已有相关报道，俞达辉等^[36]^的研究发现，肺腺癌肿瘤组织中CD45RO表达水平越高，患者的DFS及OS越长。这与我们的研究结果基质区CD45RO^+^ TILs的密度是肺腺癌的独立预后因素一致。Paulsen等^[12]^在他们的研究对列里发现CD45RO^+^ TILs的密度在肺鳞癌而非肺腺癌中具有预后意义。这与我们的结论相反，我们的研究发现肿瘤区及基质区CD45RO^+^ TILs的密度在肺鳞癌中均不具有统计学意义。这可能与研究的人群差异有关，Paulsen的人群均为欧洲人种，而我们的研究均为亚洲人种。除了人种之外，我们在研究中发现CD45RO^+^ TILs的密度与患者的吸烟情况以及肿瘤的临床分期相关，这也可能是导致研究结果不同的原因。需要扩大样本量来获得更有统计学说服力的数据。

本研究还联合CD45RO^+^ TILs密度和患者肿瘤细胞PD-L1表达水平对NSCLC患者进行生存预后分析。NSCLC生存分析显示，双标志物评分PD-L1^+^/CD45RO^-^患者组相比于其他三组有最短的OS及DFS。这一结果也比较符合当下的假说，即肿瘤高表达PD-L1促进肿瘤逃逸，而高免疫细胞浸润显示机体对肿瘤杀伤的“高敏感”状态。CD45RO^+^ TILs和PD-L1都是参与肿瘤免疫过程的重要分子。因此研究这两个标志物对未经免疫治疗患者的预后影响，可能更具有临床价值。

本研究存在一些局限性。首先，这是一项回顾性研究，样本量相对较小，随访资料的收集可能存在一些偏倚。其次，组织微阵列芯片中的芯条只是肿瘤的一小部分，我们可能需要在大组织中进行更深入的研究，挖掘更有价值的信息。

综上所述，我们的研究揭示了基质区CD45RO^+^ TILs在NSCLC及其亚组肺腺癌中是一种强有力的预后因子，是较好的OS的独立预后因素。当CD45RO^+^ TILs与肿瘤细胞表达的PD-L1联合使用时，NSCLC中PD-L1^+^/CD45RO^-^患者组相比于其他三组有最短的OS及DFS，可作为较差的预后预测因子。
